# Impacts of metabolic disruption, body mass index and inflammation on cognitive function in post-COVID-19 condition: a randomized controlled trial on vortioxetine

**DOI:** 10.1186/s12991-024-00494-1

**Published:** 2024-02-29

**Authors:** Angela T.H. Kwan, Gia Han Le, Ziji Guo, Felicia Ceban, Kayla M. Teopiz, Taeho Greg Rhee, Roger Ho, Joshua D. Di Vincenzo, Sebastian Badulescu, Shakila Meshkat, Bing Cao, Joshua D. Rosenblat, Donovan A. Dev, Lee Phan, Mehala Subramaniapillai, Roger S. McIntyre

**Affiliations:** 1 Brain and Cognition Discovery Foundation, 77 Bloor Street West, Suite 617, Toronto, ON M5S 1M2 Canada; 2 Faculty of Medicine, University of Ottawa, Ottawa, ON Canada; 3 Institute of Medical Science, University of Toronto, Toronto, ON Canada; 4 Department of Pharmacology and Toxicology, University of Toronto, Toronto, ON Canada; 5 Michael G. DeGroote School of Medicine, McMaster University, Hamilton, ON Canada; 6 Department of Psychiatry, Yale School of Medicine, New Haven, CT USA; 7 Department of Public Health Sciences, University of Connecticut School of Medicine, Storrs, USA; 8 Department of Psychological Medicine, Yong Loo Lin School of Medicine, National University of Singapore, Singapore, Singapore; 9 Institute for Health Innovation and Technology (iHealthtech), National University of Singapore, Singapore, Singapore; 10 Key Laboratory of Cognition and Personality, Faculty of Psychology, Ministry of Education, Southwest University, Chongqing, 400715 P. R. China; 11 Department of Psychiatry, University of Toronto, Toronto, ON Canada

**Keywords:** Long COVID, Vortioxetine, Cognitive function, Digit symbol substitution test (DSST), Inflammation, Metabolic dysfunction, Body mass index (BMI)

## Abstract

**Background:**

Post-COVID-19 Condition (PCC), as defined by the World Health Organization (WHO), currently lacks any regulatory-approved treatments and is characterized by persistent and debilitating cognitive impairment and mood symptoms. Additionally, metabolic dysfunction, chronic inflammation and the associated risks of elevated body mass index (BMI) have been reported. In this study, we aim to investigate the efficacy of vortioxetine in improving cognitive deficits in individuals with PCC, accounting for the interaction of metabolic dysfunction, elevated inflammation and BMI.

**Methods:**

This is a post-hoc analysis of an 8-week randomized, double-blind, placebo-controlled trial that was conducted among adults aged 18 years and older living in Canada who were experiencing WHO-defined PCC symptoms. The recruitment of participants began in November 2021 and concluded in January 2023. A total of 200 individuals were enrolled, where 147 were randomized in a 1:1 ratio to receive either vortioxetine (5–20 mg, *n* = 73) or placebo (*n* = 74) for daily treatment under double-blind conditions. The primary outcome measure was the change in the Digit Symbol Substitution Test (DSST) score from baseline to endpoint.

**Results:**

Our findings showed significant effects for time (χ^2^ = 7.771, *p* = 0.005), treatment (χ^2^ = 7.583, *p* = 0.006) and the treatment *x* time *x* CRP *x* TG-HDL *x* BMI interaction (χ^2^ = 11.967, *p* = 0.018) on cognitive function. Moreover, the between-group analysis showed a significant improvement with vortioxetine at endpoint (mean difference = 0.621, SEM = 0.313, *p* = 0.047).

**Conclusion:**

Overall, vortioxetine demonstrated significant improvements in cognitive deficits among individuals with baseline markers of metabolic dysfunction, elevated inflammation and higher BMI at endpoint as compared to placebo.

**Trial Registration:**

NCT05047952 (ClinicalTrials.gov; Registration Date: September 17, 2021).

**Supplementary Information:**

The online version contains supplementary material available at 10.1186/s12991-024-00494-1.

## Background

The World Health Organization (WHO) has reported a cumulative global count exceeding 800 million confirmed cases of coronavirus disease 2019 (COVID-19) to date [1]. Notably, a significant proportion of individuals who have recovered from acute SARS-CoV-2 infection continue to experience persistent and debilitating symptoms, a condition known as post-COVID-19 syndrome (PCC). This phenomenon is officially defined by the WHO as the presence of debilitating symptoms that occur at least three months following a confirmed COVID-19 infection and persist for a minimum of two months [2]. Evidence suggests that approximately 10–20% of individuals who have had COVID-19 meet the criteria for PCC, where disruptions in immune-inflammatory and vascular function may be contributing to the onset [3]. Many symptoms suggesting disturbances across multiple organ systems have been documented. These symptoms include cognitive impairment (e.g., *“brain fog”*), neuropsychiatric manifestations, chronic inflammation, metabolic dysfunction and increased health risks associated with an elevated BMI [i.e., overweight (25-29.9 kg/m^2^) and obese (30-39.9 kg/m^2^)] [4–7].

Cognitive impairment is a common and significant aspect of PCC—prevalent in 22% of cases, ranking second only to fatigue at 32%—which greatly impairs the quality of life and functional abilities of those affected [4, 8]. Given the high frequency and debilitating nature of this symptom, the poorly understood neurobiological mechanisms responsible for cognitive impairment in PCC along with the overall pathoetiology of PCC underscore the need to investigate these factors. This research is essential for developing strategies to prevent, intervene early and treat cognitive impairment in individuals with PCC. Currently, no treatment has proven effective and well-tolerated in a robust and large-scale randomized, double-blind, placebo-controlled trial, nor have any treatments received regulatory approval for PCC.

To inform treatment development mechanistically, it is essential to explore interventions effective against cognitive symptoms in other medical conditions while modulating relevant neurological systems (e.g., immune-inflammation, metabolic dysfunction) for PCC [9]. Vortioxetine, a multimodal antidepressant, has been shown to improve cognitive performance in both objective and subjective assessments among adults diagnosed with Major Depressive Disorder (MDD) [10]. Furthermore, vortioxetine has immunomodulatory and antioxidative properties that are relevant to the neurobiology of PCC [11]. Thus, vortioxetine was chosen as the agent in this study because it has shown beneficial effects on measures of cognition in healthy controls as well as persons with mental illness [12, 13].

Extant evidence suggests that there is a link between overweight/obesity, metabolic disruption (e.g., insulin resistance) and inflammatory factors produced by metabolically dysfunctional adipocytes. These factors are not only interrelated but also independently associated with cognitive impairment in both the general population and individuals with psychiatric and/or medical conditions (e.g., MDD) [14, 15]. It has also been documented that proxy measures of insulin resistance (e.g., increased TG-HDL ratio) are causally associated with brain-based disorders like MDD [16–18]. Moreover, a separate line of research has shown that metabolic and inflammatory-related comorbidities (e.g., obesity, type 2 diabetes, MDD) not only serve as risk factors for COVID-19 infection but also for PCC [19].

In this study, our objective was to identify clinical characteristics linked to cognitive impairment in PCC by examining the relationships between inflammation marker C-reactive protein (CRP), BMI, a proxy measure of insulin resistance (e.g., TG-HDL), and their combined effect on cognitive functioning among individuals with PCC.

## Materials and methods

### Study Design and participants

This study is a post-hoc analysis of an 8-week randomized, double-blind, flexible-dosed, placebo-controlled clinical trial that examined the efficacy of vortioxetine for the treatment of cognitive impairments in individuals with PCC. A local research ethics board (REB) approved the trial design (Advarra, Pro00055939). Guidelines of Good Clinical Practice (ICH, 1996) and the Declaration of Helsinki (WMA, 2008) were followed. The protocol and dataset presented herein originate from the primary study, which is now published (ClinicalTrials.gov number: NCT05047952) [20].

Participant recruitment took place in Canada from November 2021 to January 2023. Recruitment efforts were facilitated via media promotions (e.g., Facebook, Twitter, Instagram and print) and referrals from medical professionals. Written informed consent was required during the screening process for inclusion of eligible persons in the study.

### Randomization and masking

A preliminary pre-screen assessment was conducted by trained trial staff for individuals who expressed interest in the study. If all inclusion criteria were met, the following step was completion of an additional eligibility evaluation. Eligible participants were then randomized in a 1:1 ratio to receive either vortioxetine (5–20 mg/day) or placebo for an 8-week double-blind treatment period. Randomization was internally completed by staff members who were blinded to treatment assignments, with sequentially enrolled participants allocated to the lowest available randomization number in blocks of 10. All study personnel, including investigators, research coordinators and participants, remained blinded to treatment assignments throughout the study. Two designated, unblinded staff members were solely responsible for labeling and dispensing the investigational product and had no participant interaction. The randomization code remained unbroken for any participant throughout the study.

### Procedures

Eligible participants were included in this study if they met the following criteria: (1) aged 18 or older, (2) reside in Canada and (3) have a history of confirmed severe acute respiratory syndrome coronavirus 2 (SARS-CoV-2) infection (e.g., positive SARS-CoV-2 PCR test, rapid antigen test, serology test) or clinical diagnosis by a healthcare provider. For individuals with probable infection, a signed confirmation of a presumptive case by a healthcare provider or formal clinical diagnosis by the study physician was required. Additionally, eligible participants must exhibit WHO-defined PCC symptoms within 3 months after the initial COVID-19 diagnosis. Written informed consent must also be obtained at the screening or baseline visit. Detailed exclusion criteria can be found in the supplementary materials (Table S1).

Participants assigned to the vortioxetine group initially received a dosage of 10 mg/day during weeks 1 and 2 of the study, which was then increased to 20 mg/day from weeks 3 to 8. However, for participants aged 65 and older within the vortioxetine group, a lower dosage of 5 mg/day was given during weeks 1 and 2, with an increment to 10 mg/day from weeks 3 to 8. Down titration to the initial dose was permitted if higher doses were not tolerated.

Throughout the study, assessments occurred at baseline with subsequent evaluations at weeks 2, 4 and 8. In cases where participants chose to withdraw from the study, their evaluations were completed at the earliest possible date following their withdrawal.

### Outcome measures

The effect of vortioxetine compared to placebo on cognitive function was assessed using the Digital Symbol Substitution Test (DSST) (Pen/Paper plus Online CogState Version as part of the CogState Online Cognitive Battery). Remote participants did not complete the Pen/Paper Version of the DSST. The DSST was administered at baseline, and weeks 2 and 8.

Participant anthropometrics (e.g., weight and height) were either measured directly at the study site by research staff or self-reported at baseline. Furthermore, baseline and week 8 blood tests were performed to assess presence of inflammation (i.e., CRP) and metabolic disruption [i.e., serum cholesterol to high-density lipoprotein (HDL) ratio].

### Statistical analysis

All statistical analyses were conducted using the IBM SPSS Statistics software, version 28.0.1.1 [15], with a two-sided statistical significance level (α) set at 0.05. Descriptive statistics were presented as frequencies (%) for categorical variables and as mean [standard deviation (SD)] for normally-distributed continuous variables. For the assessment of changes in DSST total scores from baseline, an intent-to-treat (ITT) analysis (i.e., including all randomized participants) was employed.

A generalized linear model (GLM) with a poisson probability distribution was conducted to explore the correlation between cognitive function and the interaction effect of proinflammation, metabolic disruption and BMI at baseline. Furthermore, we employed a generalized estimating equations (GEE) model to evaluate the impact of inflammation, metabolic disruption and BMI on cognitive function from baseline to endpoint.

## Results

### Patient characteristics

Baseline sociodemographic, clinical characteristics and anthropometric measures of the ITT population are presented in Table 1. No statistically significant differences were observed between the treatment groups. Among the 200 participants who provided their consent, 147 were randomized to receive vortioxetine (*n* = 73) or placebo (*n* = 74).

**Table 1:** Baseline characteristics of the intent-to-treat (ITT) population (*N* = 147)

Characteristic	Placebo (*n* = 74)	Vortioxetine (*n* = 73)	p-value*
Age (Years), Mean (SD)	44.89 (12.14)	43.84 (12.35)	0.602^a^
Sex (Female), n (%)	55 (74.32)	56 (76.71)	0.736^b^
Education, n (%)			0.390^b^
< High School	0 (0)	1 (1.37)	
High School Graduate	4 (5.41)	8 (10.96)	
College/University Degree	10 (13.51)	7 (9.59)	
Associates Degree	15 (20.27)	13 (17.81)	
Bachelor’s Degree	27 (36.49)	34 (46.58)	
Graduate Degree	15 (20.27)	9 (12.33)	
Professional Degree	3 (4.05)	1 (1.37)	
Confirmed COVID Diagnosis, n (%)	59 (79.7)	57 (78.1)	0.807^b^
QIDS-SR-16 (Total Score), Mean (SD)	10.32 (4.37)	10.03 (4.33)	0.681^a^
MDD Diagnosis, n (%)	25 (33.78)	22 (30.14)	0.595^b^
FSS (Total Score), Mean (SD)	51.84 (10.20)	49.78 (10.96)	0.083^a^
Walking Days per Week, Mean (SD)	4.18 (2.53)	4.62 (2.43)	0.283^a^
Remote Assessment, n (%)	69 (93.24)	67 (91.78)	0.736^b^
Combined DSST Z-score, Mean (SD)^c^	-0.194 (0.99)	0.0531 (1.01)	0.136^a^
CRP, Mean (SD)	3.07 (3.37)	2.43 (2.96)	0.276^a^
TRG-HDL, Mean (SD)	3.62 (0.99)	5.81 (17.19)	0.376^a^
BMI, Mean (SD)	31.25 (7.49)	29.03 (9.05)	0.113^b^
Normal Weight	21.99 (2.31)	22.57 (1.52)	
Overweight	27.93 (1.40)	27.69 (1.56)	
Obese	37.99 (4.98)	40.51 (8.33)	

^a^T-test

^b^Chi-square test

^c^Combined DSST z-score defined as the equally weighted sum of the z-scores in the combined DSST (Pen/Paper plus Online CogState Version)

*Two-sided p values;

**Abbreviations**: BMI = Body Mass Index; CRP = C-Reactive Protein; DSST = Digit Symbol Substitution Test; FSS = Fatigue Severity Scale; MDD = Major Depressive Disorder; QIDS-SR16 = Quick Inventory of Depressive Symptomatology-Self-Report 16; SD = Standard Deviation; TRG-HDL = Triglyceride / HDL-Cholesterol.

### Impacts of metabolic disruption, body mass index (BMI) and inflammation on cognitive function at baseline

A GLM analysis was conducted on 147 participants to examine the impact of metabolic disruption, elevated BMI and inflammation on cognitive function in persons with PCC at baseline. In the adjusted model (i.e., sociodemographics, clinical characteristics, anthropometric measures), our results indicate that age (β = -0.016; 95% confidence interval [CI], -0.033-0.002; *p* = 0.081) and the TG-HDL *x* BMI *x* CRP interaction effect (β = -0.021; 95% CI, -0.038-(-0.003); *p* = 0.023) both had a significant negative association with performance on the DSST **(**Table 2**)**.

**Table 2:** Generalized linear model of the association between objective cognitive function and inflammation, metabolic disruption and BMI in individuals with post-COVID-19 condition

Model	B	Coefficients Standard Error	95% Confidence Interval (CI)		
			Lower	Upper	P value
**Age**	-0.016	0.0089	-0.033	0.002	0.081
**Sex**	-0.136	0.2403	-0.607	0.335	0.571
**Education**	0.102	0.0977	-0.089	0.294	0.296
**CRP**	-2.248*	0.9492	-4.108	-0.388	0.018
**TG-HDL**	-2.123**	0.7641	-3.621	-0.626	0.005
**BMI**	-0.253**	0.0972	-0.444	-0.063	0.009
**TG-HDL** ***x*** **BMI** ***x*** **CRP**	-0.021*	0.0091	-0.038	-0.003	0.023
**Suspected vs. Confirmed COVID-19**	0.176	0.2867	-0.386	0.738	0.540
**QIDS-SR-16**	0.046	0.0294	-0.011	0.104	0.117
**MDD Diagnosis**	0.163	0.2514	-0.329	0.656	0.516
**Fatigue**	-0.047**	0.0124	-0.071	-0.022	< 0.001
**Alcohol Consumption** ***(Drinks per Week)***	-0.035	0.0330	-0.100	0.030	0.289
**Marijuana Frequency**	-0.171*	0.0708	-0.310	-0.033	0.015

Dependent variable: DSST total score

*p < 0.05, **p < 0.01

### Effects of metabolic disruption, elevated body mass index (BMI) and inflammation on cognitive function: comparing vortioxetine and placebo

**Fig. 1:**
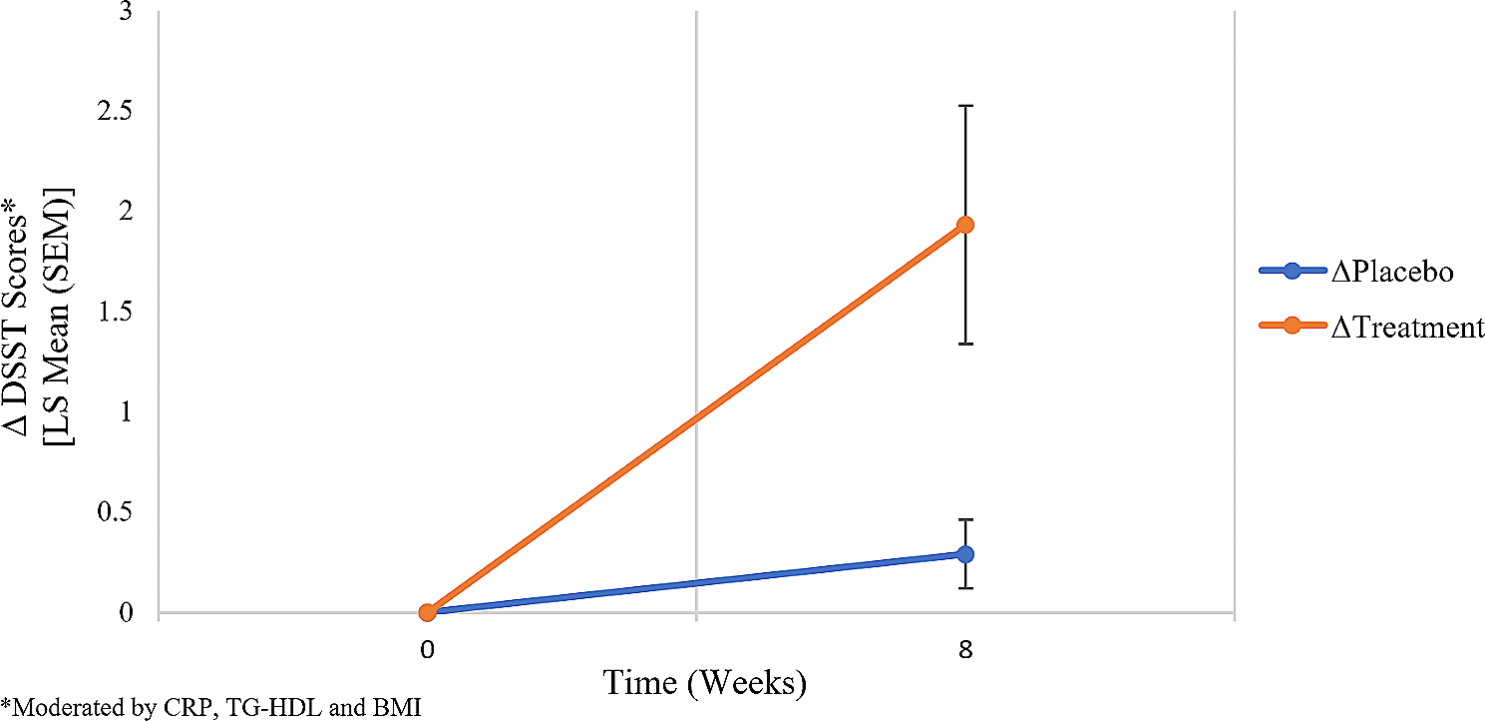
An intention-to-treat GEE analysis investigating the effects of vortioxetine (*n* = 73) versus placebo (*n* = 74) on the interplay between cognitive function with metabolic disruption, elevated BMI and inflammation in an 8-week trial. The least square (LS) mean (standard error of mean [SEM]) values are depicted for the change in DSST total scores from baseline to endpoint, using an independent covariance matrix with time as a categorical variable

An ITT GEE analysis was performed on the 147 participants who were randomized to receive either vortioxetine (*n* = 73) or placebo (*n* = 74). In the unadjusted model, significant treatment (χ^2^ = 7.583, *p* = 0.006), time (χ^2^ = 7.771, *p* = 0.005) and the treatment *x* time *x* CRP *x* TG-HDL *x* BMI interaction (χ^2^ = 11.967, *p* = 0.018) effects were observed. This suggests that DSST-measured cognitive function improved over time and at different rates between the treatment groups. Similarly, in the adjusted model (i.e., sociodemographics, clinical characteristics, anthropometric measures), significant effects were observed for treatment (χ^2^ = 4.403, *p* = 0.036), treatment *x* time (χ^2^ = 14.090, *p* < 0.001) and the treatment *x* time *x* CRP *x* TG-HDL *x* BMI interaction (χ^2^ = 12.979, *p* = 0.011) on cognitive function at the endpoint. However, there was no significant time effect (χ^2^ = 0.189, *p* = 0.664). These findings suggest that participants’ cognitive function improved at different rates within each treatment group but did not significantly improve over time **(**Fig. 1**)**.

Additionally, in the adjusted model, our findings showed a significant within-group change in DSST scores for the vortioxetine group (mean difference = 1.933, SEM = 0.594, *p* = 0.001); the placebo group, however, showed an insignificant change (mean difference = 0.289, SEM = 0.171, *p* = 0.091) **(**Table 3; Fig. 1**)**. When comparing the two groups, the between-group analysis showed a significant difference in overall change in favour of vortioxetine at the 8-week endpoint (mean difference = 0.621, SEM = 0.313, *p* = 0.047).

**Table 3:** Pairwise comparisons of the estimated marginal means based on the efficacy endpoint (Composite DSST) in the intent-to-treat population

(I) Treatment Allocation x Week	(J) Treatment Allocation x Week	Mean Difference (I-J)	Standard Error	95% Confidence Interval		
				Lower	Upper	P-value
**Adjusted Model †**						
Treatment Allocation (Placebo) x Week 8	Treatment Allocation (Placebo) x Week 0	0.289	0.171	-0.046	0.624	0.091
Treatment Allocation (Vortioxetine) x Week 8	Treatment Allocation (Vortioxetine) x Week 0	1.933^a^**	0.594	0.769	3.097	0.001
	Treatment Allocation (Placebo) x Week 0	0.910^a^**	0.307	0.307	1.512	0.003
Treatment Allocation (Vortioxetine) x Week 8	Treatment Allocation (Placebo) x Week 8	0.621^a^*	0.313	0.008	1.234	0.047

Pairwise comparisons of the estimated marginal means based on the original scale of the dependent variable (DSST total score), with moderation by inflammation, metabolic dysfunction and BMI.

a. The mean difference is significant at the 0.05 level

† Adjusted for sociodemographics, clinical characteristics, anthropometric measures and confirmed COVID-19 diagnosis

*p < 0.05, **p < 0.01

## Discussion

Herein, we observed that laboratory evidence of inflammation (i.e., CRP), insulin resistance (i.e., increased TG-HDL ratio) and elevated BMI (i.e., overweight/obesity) are highly associated with cognitive impairment in individuals with PCC. Specifically, we found that the interaction effect of CRP *x* TG-HDL *x* BMI was negatively correlated with DSST performance at baseline. This suggests that high levels of inflammation, metabolic disruption and elevated BMI are linked to more pronounced cognitive deficits in PCC. Furthermore, we observed a significant improvement in objective cognitive function over time, along with a significant between-group difference, at the endpoint among vortioxetine-treated participants who exhibited high baseline markers of inflammation, metabolic disruption and elevated BMI.

These findings enhance our understanding of cognitive impairment in PCC and align with prior research on the risks of metabolic disorders, high BMI and conditions characterized by inflammation in relation to COVID-19 susceptibility and the development of PCC [21–23]. Furthermore, our data are consistent with a compelling body of evidence showing that disruptions in inflammation, metabolic function and obesity hazardously affect brain health, increasing susceptibility to central nervous system and psychiatric disorders [14, 17, 24–27]. Moreover, our study supports the finding that individuals with obesity tend to experience a wider range of PCC symptoms compared to those without obesity. Adipocytes and immune cells, which act as inflammatory partners in promoting and perpetuating inflammation, also accompany the metabolic syndrome in PCC [28–32]. Thus, it can be conjectured that individuals living with PCC may manifest disturbances in objective cognitive function due to baseline inflammation, metabolic disruption and a high BMI status.

It is hypothesized that inflammatory and metabolic changes affect neuronal and glial integrity, as evidenced by alterations in functional connectivity within and between neural circuits associated with cognitive functions [33–35]. It can further be conjectured that interventions that prevent or reduce inflammation or metabolic disruption in persons with PCC-related cognitive impairment may potentially be therapeutic for these individuals. Future research should investigate the relationship between inflammatory markers, BMI, oxygenated hemoglobin levels and DSST performance using functional neuroimaging [36].

Our study has several methodological limitations that could impact the interpretation and inference of our data. First, this is a post-hoc analysis of data originally collected in a primary study. The investigation of the relationship between objective cognitive function with elevated inflammation, metabolic dysfunction and BMI was not pre-determined as the primary outcome measure in the study protocol. As a result, our methodological approach does not allow us to establish cause-and-effect relationships or the temporal sequence of events comprehensively. Second, while we excluded other medical conditions (e.g., MDD) as primary causes of cognitive deficits, it remains possible that these symptoms could be linked to participants’ previous or undiagnosed medical issues. Third, we only used one variable (e.g., TG-HDL) as a proxy measure for metabolism and CRP for inflammation, which is a nonspecific marker that can be elevated for reasons unrelated to disease (e.g., smoking, drinking, trauma). Fourth, some covariates that may have been contributory were not adjusted for (e.g., medical comorbidity, premorbid cognitive status). Furthermore, it remains unclear whether individuals in our sample, who are living with PCC, experienced alterations in their inflammation and metabolic markers preceding their COVID-19 infection or onset of PCC. Additionally, our participant sample exhibited heterogeneity in regard to the number and severity of acute COVID-19 symptoms, past COVID-19 infections, vaccination history and the duration of persistent PCC symptoms.

Overall, we would conceptualize our results as hypothesis-generating rather than hypothesis-confirming, thus further research such as neuroimaging studies is necessary to fully provide substantial evidence to support our thesis. Nonetheless, our findings are in keeping with the conceptual framework documenting a robust association between cognitive function with elevated inflammation, metabolic dysfunction and high BMI. Our sample of individuals living with PCC was well characterized with cognitive measures evaluated using a valid and reliable measure.

## Conclusion

In summary, our post-hoc analysis demonstrates a significant association between elevated inflammation, metabolic disruption, BMI with reduced cognitive function in individuals with PCC. We hypothesize that individuals with these factors may positively respond to vortioxetine treatment, potentially showing a distinct treatment response profile. Larger studies with predefined variables are required to validate these hypotheses. If confirmed, these results could open promising avenues for therapeutic interventions targeting inflammation and metabolism, aiming to alleviate symptoms and reduce the overall disease burden. In addition, clinicians providing care to persons living with PCC should be vigilant for the possibility of and monitor for metabolic disruption and increased BMI.

### Electronic supplementary material

Below is the link to the electronic supplementary material.

Supplementary Material 1

## Data Availability

The datasets generated and/or analyzed during the current study are not publicly available due to protection of patient information but are available from the corresponding author, R.S.M, upon reasonable request and will be anonymized.

## Declarations

Felicia Ceban, Kayla M. Teopiz and Mehala Subramaniapillai have received fees from Braxia Scientific Corp.

Dr. Taeho Greg Rhee was supported in part by the National Institute on Aging (NIA) (#R21AG070666; R21AG078972), National Institute of Mental Health (#R21MH117438), National Institute on Drug Abuse (#R21DA057540) and Institute for Collaboration on Health, Intervention, and Policy (InCHIP) of the University of Connecticut. Dr. Rhee serves as a review committee member for Patient-Centered Outcomes Research Institute (PCORI) and Substance Abuse and Mental Health Services Administration (SAMHSA) and has received honoraria payments from PCORI and SAMHSA. Dr. Rhee has also served as a stakeholder/consultant for PCORI and received consulting fees from PCORI. Dr. Rhee serves as an advisory committee member for International Alliance of Mental Health Research Funders (IAMHRF). Dr. Rhee is currently a co-Editor-in-Chief of *Mental Health Science* and has received honorarium payments annually from the publisher, John Wiley & Sons, Inc.

## Abbreviations

BMI: Body Mass Index
COVID-19: Coronavirus Disease 2019
CRP: C-reactive Protein
DSST: Digit Symbol Substitution Test
GEE: Generalized Estimating Equations
GLM: Generalized Linear Model
HDL: High-Density Lipoprotein
ITT: Intent-to-treat
MDD: Major Depressive Disorder
PCC: Post-COVID-19 Condition
REB: Research Ethics Board
SARS-CoV-2: Severe Acute Respiratory Syndrome Coronavirus 2
TG-HDL: Triglyceride/High-Density Lipoprotein
WHO: World Health Organization
