# Supplementary material for: Impacts of metabolic disruption, body mass index and inflammation on cognitive function in post-COVID-19 condition: a randomized controlled trial on vortioxetine
Source: Ann Gen Psychiatry. 2024 Feb 29;23:10. doi: 10.1186/s12991-024-00494-1 (PMC10905871; doi:10.1186/s12991-024-00494-1)
Supplement: Supplementary file 1 — Supplementary Material 1 [file 12991_2024_494_MOESM1_ESM.docx]

**Supplementary Material**

**Table S1.** Exclusion criteria

| - Current symptoms were better explained by symptoms of major depressive disorder or bipolar disorder; - Symptoms were fully explained by pre-existing conditions that may cause cognitive impairment or symptoms similar to those seen in PCC [e.g., attention deficit/hyperactivity disorder (ADHD), major neurocognitive disorder, schizophrenia, chronic fatigue syndrome (CFS)/encephalitis meningitis (EM), as assessed by Mini International Neuropsychiatric Interview (M.I.N.I.) 7.0.2]; - Known intolerance to vortioxetine and/or prior trial of vortioxetine with demonstrated inefficacy; - Current alcohol and/or substance use disorder, as confirmed by the M.I.N.I. 7.0.2; - Presence of comorbid psychiatric disorder that is a primary focus of clinical concern, as confirmed by the M.I.N.I. 7.0.2; - Previous history of mania/hypomania; - Taking medications approved and/or employed off-label for cognitive dysfunction (e.g., psychostimulants); - Any medication for a general medical disorder that may affect cognitive function (as per clinical judgment); - Use of benzodiazepines within 12 hours of cognitive assessments; - Consumption of alcohol within eight hours of cognitive assessments; - Any physical, cognitive, or language impairments sufficient to adversely affect data derived from cognitive assessments; - Diagnosed reading disability or dyslexia; - Clinically significant learning disorder by history; - Treatment with electroconvulsive therapy (ECT) in the last 6 months; - History of moderate or severe head trauma (e.g., loss of consciousness for > 1 hour), other neurological disorders, or unstable systemic medical diseases that are likely to affect the central nervous system (as per clinical judgment); - Pregnant and/or breastfeeding; received investigational agents as part of a separate study within 30 days of the screening visit; - Actively suicidal/presence of suicidal ideation or evaluated as being at suicide risk (as per clinical judgment); - Currently receiving treatment with monoamine oxidase inhibitor (MAOI) antidepressants, antibiotics such as linezolid or intravenous methylene blue; - Previous hypersensitivity reaction to vortioxetine or any components of the formulation; - Previously reported angioedema in persons treated with vortioxetine; - Serotonin syndrome; - Abnormal bleeding; - Angle closure glaucoma; - Hyponatremia; - Moderate hepatic impairment; - Active seizure disorder/epilepsy that is not controlled by medication (as per clinical judgment); - Presence of any unstable medical conditions; - Inability to follow study procedures; - And inability to give informed consent. |
| --- |
